# Complications after partial nephrectomy: robotics overcomes open surgery and laparoscopy: the PMSI French national database

**DOI:** 10.1186/s12894-023-01322-6

**Published:** 2023-09-15

**Authors:** Antoine Bic, Charles Mazeaud, Julia Salleron, Aurélie Bannay, Beverley Balkau, Clément Larose, Jacques Hubert, Pascal Eschwège

**Affiliations:** 1 Service d’Urologie CHRU Nancy, Site Brabois, Nancy, 54000 France; 2 Department of Urology, Nancy University Hospital, Avenue de Bourgogne, Vandoeuvre Cedex, 54511 France; 3 Département de Biostatistiques, Institut de Cancérologie de Lorraine, 6 avenue de Bourgogne CS 30519, Vandoeuvre-lès-Nancy Cedex, 54519 France; 4 Service d’Évaluation et Information Médicales, CHRU Nancy, Nancy, France; 5 Épidémiologie Clinique, Centre de Recherche en Épidémiologie et Santé des Populations, Institut National de la Santé et de la Recherche Médicale U1018, Université Paris-Saclay, USVQ, Université Paris-Sud, Villejuif, F-94807 France

**Keywords:** Renal cancer, Nephron-sparing, Partial nephrectomy, Robot, Laparoscopy, Open surgery, PMSI

## Abstract

**Purpose:**

To evaluate three partial nephrectomies (PN) procedures: open (OPN), standard laparoscopy (LPN), and robot-assisted laparoscopy (RAPN), for the risk of initial complications and rehospitalization for two years after the surgery.

**Materials and methods:**

From the French national hospital database (PMSI-MCO), every hospitalization in French hospitals for renal tumor PN in 2016–2017 were extracted. Complications were documented from the initial hospitalization and any rehospitalization over two years. Chi-square and ANOVA tests compared the frequency of complications and length of initial hospitalization between the three surgical procedures. Relative risks (RR) and 95% confidence intervals were computed.

**Results:**

The 9119 initial hospitalizations included 4035 OPN, 1709 LPN, and 1900 RAPN; 1475 were excluded as the laparoscopic procedure performed was not determined. The average length of hospitalization was 8.1, 6.2, and 4.5 days for OPN, LPN, and RAPN, respectively. Compared to OPN, there were fewer complications at the time of initial hospitalization for the mini-invasive procedures: 29% for OPN vs. 20% for LPN (0.70 [0.63;0.78]) and 12% for RAPN (RR=0.43, 95%CI [0.38;0.49]). For RAPN compared to LPN, there were fewer haemorrhages (RR=0.55 [0.43;0.72]), anemia (0.69 {0.48;0.98]), and sepsis (0.51 [0.36;0.71]); during follow up, there were fewer urinary tract infections (0.64 [0.45;0.91]) but more infectious lung diseases (1.69 [1.03;2.76]). Over the two-year postoperative period, RAPN was associated with fewer acute renal failures (RR=0.73 [0.55;0.98]), renal abscesses (0.41 [0.23;0.74]), parietal complications (0.69 [0.52;0.92]) and urinary tract infections (0.54 [0.40;0.73]) than for OPN.

**Conclusions:**

Conservative renal surgery is associated with postoperative morbidity related to the surgical procedure fashion. Mini-invasive procedures, especially robot-assisted surgery, had fewer complications and shorter hospital lengths of stay

## Introduction

Conservative surgical management represents the standard of care for small renal tumors [1]. For several years we have been witnessing the development of the minimally invasive approach, laparoscopic (LPN) or robotic surgery (RAPN), which offers the same survival and oncological outcomes and reduces the perioperative morbidity [2–7].

With the spread of robotics techniques, the rate of PN procedures has increased [8–11]. Robotic assistance has allowed us to expand the limit of nephron-sparing-surgery [12] and to manage more complex tumours with a shorter learning curve [13].

Nonetheless, the benefits of RAPN remain controversial. The data in the literature are heterogeneous [2–7, 14, 15] and present several limitations (monocentric studies, selective inclusion criteria, small sample size, only expert centres). The last report on robot-assisted nephrectomy from the French National Authority for Health in 2019 [16] stated that available data are lacking and do not allow a conclusion about a clinical improvement compared to the open or laparoscopic approach.

This study retrieved data from the French national hospital discharge database and associated hospital stays in general medicine, surgery, and obstetric medical facilities (Programme de Médicalisation des Systèmes d’Information: PMSI). Declarations in this database are mandatory for all medical activities in every public or private French hospital and are the calculation basis for billing by the national healthcare insurance system. Extensive hospitalization data are registered for each medical unit consulted, including administrative information (age, gender, length of stay) and medical information such as the primary diagnosis and associated significant diagnoses, comorbidity or complication that correspond to all conditions that could impact the length of stay. Hospitalizations are classed under a *Groupe Homogène de Malades* (GHM) similar to the American model: *Diagnosis Related Group* (DRG), with both medical and cost information corresponding to the billing. The data are anonymous and accessible to collect the largest French database of partial nephrectomy (PN) clinical outcomes.

Our study assessed the three procedures of partial nephrectomy for kidney cancer in the French population, especially the risk of complications associated with the initial hospitalization and the rehospitalizations over two years.

## Patients, materials and methods

### Data collection

All hospitalizations in France between January 1, 2016, and December 31 2017, with one of the PN codes from the *Classification Commune des Actes Médicaux* (CCAM) were extracted retrospectively from the PMSI database. We used an additional filter for diagnosing renal cancer, C64, from the International Classification of Diseases 10th edition (ICD-10). Our local Department of Medical Information controlled coding discrepancies.

### Procedures used for partial nephrectomy

The three procedures were identified with respective codes for surgical and interventional procedures of CCAM:

- OPN had its CCAM codes: laparotomy (JAFA019 / JAFA030) and lumbotomy (JAFA008 / JAFA024).
- RAPN did not have a specific CCAM code during the inclusion period. We have identified the official list of centres with surgical robots in France. The RARP group was composed of all these centres reporting LPN codes. We excluded centres that potentially overestimated the rate of complications associated with the learning curve of the robotic technique. All centres were contacted to include only those using the robot systematically for each PN for at least five years. We selected the centers, not the surgeons, and simply validated the current use of the robot (without regard to individual experience).
- LPN (JAFC005) included all patient hospitalizations after removal of hospitals using the RAPN technique. LPN complications were from hospitals using the LPN technique exclusively.

### Complications

The “*Résumé de Sortie Standardisé*” (RSS) was extracted from the PMSI. The primary and secondary diagnoses were retrieved with the ICD-10 codes. Each complication was grouped according to two periods:

- The initial hospitalization.
- Rehospitalizations any causes, during the two years following the initial operation, for complications that were not present at the initial hospitalization.

Complications were classified with the Clavien scale. We separated into moderate complications (Clavien 1 and 2) and severe complications (Clavien ≥ 3).

### Statistical analysis

Variables were described by frequency (percentage) and mean ± standard deviation. Chi-square or Fisher tests were performed to compare the frequencies of complications and ANOVA for the mean durations of hospitalization for the three procedures. If a statistically significant difference existed between the three procedures studied, they were compared two by two, and a Bonferroni correction was applied. The relative risks (RRs) and their 95% confidence intervals were computed. Statistical analyses used SAS, version 9.4 (SAS Institute Inc., Cary, NC, USA). The level of statistical significance was set at 5%.

## Results

### Study population

In 2016 and 2017, 11,698 hospitalizations had a PN code from 260 profit and 183 non-profit establishments. After filtering for the diagnosis of renal cancer and controlling for coding discrepancies, 9119 hospitalizations for PN remained for study: 4035 OPN, 1900 RAPN and 1709 LPN. We excluded 1475 hospitalizations because the robotic or laparoscopic nature of the procedures was not homogeneous in the centres or the robot was being used for less than five years.

In 2017, they were 83 centres that had the surgical robot, 36 in-profit establishments (13%) and 47 non-profit establishments (25%). The RAPN group was composed only of PN from the 25 centres using exclusively RAPN for more than five years.

Open surgery was more frequent in the centres using standard laparoscopy than in centres using robotic surgery, 63% vs. 21%.

### Data on the initial hospitalization

The average hospital stay was 8.1 days for OPN, 6.2 for LPN and 4.5 for RAPN, with RAPN being shorter than OPN (p = 0.018) (Table 1). There was no significant difference between for-profit and non-profit hospitals.

**Table 1:** Characteristics of patients operated by partial nephrectomy procedures, their length of initial hospitalization and type of hospital: from the French national hospital database (PMSI-MCO)

	Partial Nephrectomy Procedure		
	Open (OPN)	Robot-assisted (RAPN)	Laparoscopy (LPN)
	n = 4035	n = 1900	n = 1709
Age (years)	62 ± 13	60 ± 13	62 ± 12
Male	2738 (68%)	1313 (69%)	1190 (70%)
Female	1297 (32%)	587 (31%)	519 (30%)
Type of hospital			
Profit	1826 (45)	263 (14)	1042 (61)
Non-profit	2209 (55)	1637 (86)	667 (39)
Length of stay			
All	8.1 ± 5.3	4.5 ± 3.2	6.2 ± 3.7
Profit	8,2 ± 4,8	5,3 ± 2,5	6,2 ± 3,0
Non-Profit	8.1 ± 5,7	4,4 ± 3,3	6,2 ± 4,5

Data are mean ± SD or n (%)

Among the 7644 patients in our study, no deaths were reported during the initial hospitalization (Table 2). Compared to OPN, there were fewer patients with complications than for the mini-invasive procedures: one or more complications were reported for 1173 (29%) patients for OPN vs. 236 (12%) for RAPN (RR = 0.43) and 350 (20%) for LPN (RR = 0.70) (p < 0.001).

**Table 2:** Complications at initial hospitalization for partial nephrectomy, national French data from the PMSI

	OPN	RAPN	LPN	Relative risk and 95% confidence interval		
	N = 4035	N = 1900	N = 1709	RAPN vs. OPN	LPN vs. OPN	RAPN vs. LPN
**CLAVIEN I/II**	**1303 (32.29)**	**262 (13.79)**	**331 (19,37**	**0.43 [0.38;0.48]** ^**1**^	**0.60 [0.54;0.67]** ^**1**^	**0.71 [0.61;0.83]** ^**1**^
Acute renal failure	200 (4.96)	38 (2.0)	39 (2.28)	0.40 [0.29;0.57] ^1^	0.46 [0.33;0.65] ^1^	0.88 [0.56;1.36]
Surgical incision complications	71 (1.76)	10 (0.53)	10 (0.59)	0.30 [0.15;0.58] ^1^	0.33 [0.17;0.64] ^1^	0.9 [0.38;2.16]
Anaemia	243 (6.02)	52 (2.74)	68 (3.98)	0.45 [0.34;0.61] ^1^	0.66 [0.51;0.86] ^1^	0.69 [0.48;0.98] ^1^
Urinary tract infection	288 (7.14)	75 (3.95)	75 (4.39)	0.55 [0.43;0.71] ^1^	0.61 [0.48;0.79] ^1^	0.90 [0.66;1.23]
Renal abscess	11 (0.27)	1 (0.01)	3 (0.18)	0.19 [0.02;1.49]	0.64 [0.18;2.31]	0.30 [0.03;2.88]
Venous thromboembolic disease	51 (1.26)	9 (0.47)	17 (0.99)	0.37 [0.18;0.76] ^1^	0.79 [0.46;1.36]	0.48 [0.21;1.07]
Infectious lung diseases	77 (1.91)	15 (0.79)	17 (0.99)	0.41 [0.24;0.72] ^1^	0.52 [0.31;0.88] ^1^	0.79 [0.4;1.58]
Surgical incision complications	71 (1.76)	10 (0.53)	10 (0.59)	0.30 [0.15;0.58] ^1^	0.33 [0.17;0.64] ^1^	0.9 [0.38;2.16]
Sepsis	291 (7.21)	52 (2.74)	92 (5.38)	0.38 [0.28;0.51] ^1^	0.75 [0.59;0.94] ^1^	0.51 [0.36;0.71] ^1^
**CLAVIEN ≥ III**	**464 (11.50)**	**104 (5.47)**	**160 (9.36)**	**0,48 [0.39;0.58]** ^**1**^	**0.81 [0.69;0.97]** ^**1**^	**0,58 [0.46;0.74]** ^**1**^
Haemorrhage	365 (9.05)	88 (4.63)	143 (8.37)	0.51 [0.41;0.64] ^1^	0.93 [0.77;1.11]	0.55 [0.43;0.72] ^1^
Peritonitis	20 (0.50)	4 (0.21)	6 (0.35)	0.42 [0.15;1.24]	0.71 [0.28;1.76]	0.60 [0.17;2.12]
False aneurysm	17 (0.42)	3 (0.16)	1 (0.06)	0.37 [0.11;1.28]	0.14 [0.02;1.04]	2.70 [0.28;25.92]
Renal fistula	35 (0.87)	7 (0.37)	10 (0.59)	0.42 [0.19;0.95] ^1^	0.67 [0.33;1.36]	0.63 [0.24;1.65]
Traumatic pneumothorax	27 (0.67)	2 (0.11)	0 (0)	0.16 [0.04;0.66] ^1^	-	-
Mortality	0	0	0	-	-	-

Data shown are n (%) and relative risk (RR) with 95% CI

OPN: Open partial nephrectomy; RAPN: Robot-assisted partial nephrectomy; LPN: Laparoscopic partial nephrectomy

^1^ Significantly decreased risk

In comparison to OPN, RAPN had fewer cases of haemorrhage, postoperative anaemia, renal fistula, acute renal failure, surgical incision complications, urinary tract infection, venous thromboembolic disease, postoperative occlusion, infectious lung disease, pneumothorax disease and sepsis with RRs from 0.16 to 0.55.

For LPN compared with OPN, there were fewer patients with postoperative anaemia, acute renal failure, surgical incision complications, urinary tract infection, postoperative occlusion, infectious lung disease and sepsis with RR ranging from 0.33 to 0.75 (all p < 0.05).

There were fewer cases of haemorrhage, postoperative anaemia and sepsis with RAPN than with LPN (RR 0.55, 0.69 and 0.51, respectively).

### Data on rehospitalizations during the two years following the initial hospitalization

Overall, 13% of patients had one or more complications leading to rehospitalization, with no difference between procedures (Table 3) and, in particular, no difference in overall mortality (related or not to a surgical procedure).

**Table 3:** Complications during the two years after the initial hospitalization for partial nephrectomy: national French data from the PMSI

	OPN	RAPN	LPN	Relative risk and 95% confidence interval		
	N = 4035	N = 1900	N = 1709	RAPN vs. OPN	LPN vs. OPN	RAPN vs. LPN
**CLAVIEN I/II**	**1306 (32.37)**	**445 (23.42)**	**432 (25.28)**	**0.72 [0.66;0.79]** ^**1**^	**0.8 [0.73;0.88]** ^**1**^	**0.93 [0.83;1.04]**
Acute renal failure	174 (4.31)	60 (3.16)	60 (3.51)	0.73 [0.55;0.98] ^1^	0.81 [0.61;1.09]	0.9 [0.63;1.28]
Surgical incision complications	193 (4.78)	63 (3.32)	59 (3.45)	0.69 [0.52;0.92] ^1^	0.72 [0.54;0.96] ^1^	0.96 [0.68;1.36]
Anaemia	110 (2.73)	40 (2.11)	47 (2.75)	0.77 [0.54;1.1]	1.01 [0.72;1.41]	0.77 [0.50;1.16]
Urinary tract infection	201 (4.98)	51 (2.68)	72 (4.21)	0.54 [0.40;0.73] ^1^	0.85 [0.65;1.1]	0.64 [0.45;0.91] ^1^
Renal abscess	67 (1.66)	13 (0.68)	17 (0.99)	0.41 [0.23;0.74] ^1^	0.60 [0.35;1.02]	0.69 [0.34;1.41]
Venous thromboembolic disease	69 (1.71)	22 (1.16)	27 (1.58)	0.68 [0.42;1.09]	0.92 [0.59;1.44]	0.73 [0.42;1.28]
Infectious lung diseases	92 (2.28)	45 (2.37)	24 (1.40)	1.04 [0.73;1.48]	0.62 [0.39;0.96] ^1^	1.69 [1.03;2.76] ^2^
Surgical incision complications	193 (4.78)	63 (3.32)	59 (3.45)	0.69 [0.52;0.92] ^1^	0.72 [0.54;0.96] ^1^	0.96 [0.68;1.36]
Sepsis	207 (5.13)	88 (4.63)	67 (3.92)	0.90 [0.71;1.15]	0.76 [0.58;1]	1.18 [0.87;1.61]
**CLAVIEN ≥ III**	**297 (7.36)**	**123 (6.47)**	**77 (4.51)**	**0.88 [0.72;1.08]**	**0.61 [0.48;0.78]** ^**1**^	**1.44 [1.09;1.90]**
Haemorrhage	163 (4.04)	72 (3.79)	47 (2.75)	0.94 [0.71;1.23]	0.68 [0.49;0.94] ^1^	1.38 [0.96;1.98]
Peritonitis	38 (0.94)	14 (0.74)	5 (0.29)	0.78 [0.42;1.44]	0.31 [0.12;0.79] ^1^	2.52 [0.91;6.98]
False aneurysm	36 (0.89)	21 (1.11)	13 (0.76)	1.24 [0.73;2.12]	0.85 [0.45;1.60]	1.45 [0.73;2.89]
Renal fistula	59 (1.46)	16 (0.84)	12 (0.70)	0.58 [0.33;1]	0.48 [0.26;0.89] ^1^	1.2 [0.57;2.53]
Traumatic pneumothorax	1 (0.02)	0 (0.0)	0 (0.0)	-	-	-
Mortality	0	0	0	-	-	-

Data shown are n (%) and relative risk (RR) with 95% CI

OPN: Open partial nephrectomy; RAPN: Robot-assisted partial nephrectomy; LPN: Laparoscopic partial nephrectomy

^1^ Significant decrease risk; ^2^ Significant increase risk

Compared to OPN, there were fewer acute renal failures, renal abscesses, surgical incision complications, and urinary tract infections with RAPN. In addition, we found fewer rehospitalizations for peritonitis with LPN than in those treated by OPN: 0.3% vs. 0.9%.

When comparing RAPN to LPN, there were fewer urinary tract infections for the robotic procedure (2.7% vs. 4.2%, RR = 0.64) but more infectious lung diseases (2.4% vs. 1.4%, RR = 1.69).

## Discussion

For the first time, the French national PMSI database was used to describe the complications associated with the initial hospitalization and rehospitalization after partial nephrectomy, according to the surgical procedure used.

With a population of 7644 patients operated for PN with two years of follow-up, our results provide, to our knowledge, the most extensive retrospective comparative study. It also provides an overview of PN surgery in France.

Mini-invasive procedures were used more than open surgery: 5084 RARP & LPN vs. 4035 OPN. Even if we don’t have the complete data for all mini-invasive techniques, we found that the OPN procedure was more often performed in profit hospitals. In contrast, the RAPN procedure was dominant in non-profit hospitals.

Academic centres have a high surgical volume and represent many non-profit hospitals. The proportion of robotic surgery is also twice as high. These two elements explain in part this difference.

### Advantages of mini-invasive procedures

Equivalence in terms of overall and specific long-term survival was already established in the literature [2, 4]. Only improving postoperative pain and reducing hospital stays have been formerly demonstrated [17].

Our PMSI data confirm they performed better than the open procedure for almost all complications during the initial hospitalization and reduced the length of stay. Many reference studies found similar results [5, 7].

During the two years postoperative, the open procedure was associated with more readmissions for acute renal failure, renal abscesses, surgical incision complications, and urinary tract infections than robotic surgery. Similar results are found in the literature: Camp and al. [18] also showed that OPN was associated with surgical incision complications.

Moreover, we hypothesize that the higher rate of lung infection in the RAPN group at two years reflects a more fragile population in the robotic group than an impact of surgical technique on lung condition.

Another dreaded urologic complication is false aneurysms. We found no difference between procedures. Their frequency is variable and inconsistent in published studies. The meta-analysis by Jain and al. [19] found more false aneurysms following mini-invasive surgery (2.0% vs. 1.0%). In contrast, in the series published by Peyronnet and al. [5], the robotic procedure had fewer pseudo aneurysms than the open procedure (3.6% vs. 5.5%).

In the case of false aneurysms, there was no specific ICD-10 code. We resorted to the code I722: Aneurysm of renal arteries. The lack of precision could explain the differences found in our study (1.3% for OPN) and the literature (5.5%). [5].

### Comparison of the mini-invasive procedures RAPN and LPN

Standard laparoscopy has many advantages over OPN, but robotics provides further advantages like 3D vision, increased manoeuvrability with 7 degrees of liberty, and the absence of the pivot effect. These benefits facilitate dissection and control of haemostasis and urostasis, which may explain why we found fewer haemorrhages with robotic surgery, in agreement with Bravi et al. [20], who showed that there was less blood loss with robotic surgery.

The advantages of the robotic approach allowed surgeons to maximise renal functional preservation, promoting a faster tumour dissection and a smoother renorrhaphy.

To optimise functional results, new approaches to limit hypoperfusion have been described. Surgery without clamping has shown feasibility and safety in the literature, even for complex tumours [21, 22]

The continuous efforts of expert urological surgeons in reference centres have aimed to exploit the possibilities of robotic assistance to maximise functional results as much as possible. Recent studies evaluated “Sutureless Purely Off-Clamp Robot-Assisted Partial Nephrectomy” and appeared to increase the odds of achieving the trifecta [23].

Robotic surgery can significantly limit the risk of mild or severe complications during initial hospitalization.

After two years, the benefit is no longer present; we assume that short-term complications can easily be attributed to the surgical technique. But in the longer term, confounding factors appear, and complications like pulmonary infection are more related to the patient’s condition.

We agree with the study by Achit and al [24] who compared the follow-up of four procedures for total nephrectomy in living donors where there was no difference in clinical outcomes between LPN and RAPN three months after intervention. [24].

Specific complications like postoperative urinary fistulas were rare, despite the large population in our study. No difference was seen between the two mini-invasive procedures, with 0.6% for LPN and 0.4% for RAPN, in agreement with the literature [20]. This observation can be due to the study’s design only including expert RAPN surgeons with several years of experience in robotic surgery. Zargar and al.^25^ showed that one of the main risk factors for urinary fistula was the lack of surgeon skills.

### Limits and perspectives

Our study has some limitations, given its original design. However, using data from the PMSI enables studies with large numbers of patients with better external validity, but the database was designed for medical-economic use.

#### Number of complications underestimation

In the literature, the percentages with venous thromboembolic disease range from 1 to 3.9% for OPN and RAPN and 1.1–4.2% for LPN. [26] Complications occurring out of the hospital and treated out of hospital will not be recorded in the PMSI if the patient has not been re-hospitalized. One way to correct this bias will be a study with a chain to the SNIIRAM (Système National d’Informations Inter-Régimes de l’Assurance Maladie), another database providing access to all ambulatory medical data.

#### No data on the severity conversion rate

Laparoscopic conversion to open surgery rate is not available with PMSI data. Conversions were therefore scored as laparotomy in our study. We couldn’t identify this per-operative issue. However, the literature reported a low range of around 0,7 to 5% [27], which did not significantly impact our results.

#### Few data on tumour complexity

We did not have the RENAL score that would have enabled an evaluation of the complexity of the tumour. Comparing the number of cases where open surgery was resorted to for RAPN and LPN, there were more OPNs in the LPN. The LPN tumours were probably more complex, and the surgeons resorted to open surgery in this case.

#### Possible biases of registration

Billing and reimbursement of hospitalization costs use PMSI data and depend on the GHM in which they are classified. Complications and comorbidities that receive higher refund rates could be more easily declared than those that do not, as Georgescu [28] § has shown and described two types of up-coding: altering data to optimize reimbursement or creating false comorbidities or diagnoses.

#### Perspectives

The original design of this study showed the complete data of a whole country’s population, which is the most usual way to reach firm and validated results. We avoided any selection bias to describe the effective rate of complications in a daily care situation among public and private hospitals.

In the future, we can draw various data collections applicable to patients in our population and expand our knowledge. The definition of many complications needs to be optimized for severity coding to improve accuracy for epidemiology and medico-economic use.

## Conclusions

This study is the first to evaluate the outcomes of partial nephrectomy in the entire French population. We confirm that RAPN results in a shorter hospital stay and fewer complications after the initial procedure than OPN. In addition, the robot had better results for minor and major complications than laparoscopy. Robotic technology makes it possible to treat patients with a quality of care superior to other techniques.

## Data Availability

All data generated or analyzed during this study are included in this published article.
